# The Effects of Air Pollution on Mortality in Socially Deprived Urban Areas in Hong Kong, China

**DOI:** 10.1289/ehp.10850

**Published:** 2008-07-09

**Authors:** Chit-Ming Wong, Chun-Quan Ou, King-Pan Chan, Yuen-Kwan Chau, Thuan-Quoc Thach, Lin Yang, Roger Yat-Nork Chung, Graham Neil Thomas, Joseph Sriyal Malik Peiris, Tze-Wai Wong, Anthony Johnson Hedley, Tai-Hing Lam

**Affiliations:** 1 Department of Community Medicine, School of Public Health, The University of Hong Kong, Hong Kong, China; 2 School of Public Health and Tropical Medicine, Southern Medical University, China; 3 Department of Microbiology, The University of Hong Kong, Hong Kong, China; 4 Department of Community and Family Medicine, Chinese University of Hong Kong, Hong Kong, China

**Keywords:** air pollution, case-only approach, deprivation, effect modification, Hong Kong, mortality, time-series analysis

## Abstract

**Background:**

Poverty is a major determinant of population health, but little is known about its role in modifying air pollution effects.

**Objectives:**

We set out to examine whether people residing in socially deprived communities are at higher mortality risk from ambient air pollution.

**Methods:**

This study included 209 tertiary planning units (TPUs), the smallest units for town planning in the Special Administrative Region of Hong Kong, China. The socioeconomic status of each TPU was measured by a social deprivation index (SDI) derived from the proportions of the population with *a*) unemployment, *b*) monthly household income < US$250, *c*) no schooling at all, *d*) one-person household, *e*) never-married status, and *f* ) subtenancy, from the 2001 Population Census. TPUs were classified into three levels of SDI: low, middle, and high. We performed time-series analysis with Poisson regression to examine the association between changes in daily concentrations of ambient air pollution and daily number of deaths in each SDI group for the period from January 1996 to December 2002. We evaluated the differences in pollution effects between different SDI groups using a case-only approach with logistic regression.

**Results:**

We found significant associations of nitrogen dioxide, sulfur dioxide, particulate matter with aerodynamic diameter < 10 μm, and ozone with all nonaccidental and cardiovascular mortality in areas of middle or high SDI (*p* < 0.05). Health outcomes, measured as all nonaccidental, cardiovascular, and respiratory mortality, in people residing in high SDI areas were more strongly associated with SO_2_ and NO_2_ compared with those in middle or low SDI areas.

**Conclusions:**

Neighborhood socioeconomic deprivation increases mortality risks associated with air pollution.

There is ample evidence that air pollution is a health hazard both in developed (Samet et al. 2000) and developing countries [Health Effects Institute (HEI) 2004]. Although all individuals are exposed to some level of air pollution, those who are already in poor health (Bateson and Schwartz 2004; Sunyer et al. 2000) and those who are socially disadvantaged (Forastiere et al. 2006; Jerrett et al. 2004; Neidell 2004) are most strongly affected. Globalization has resulted in the shifting of industries notorious for their pollution from wealthier to poorer areas, where costs of production are cheaper and environmental regulations are less stringent (Pulido 2000). Disparities in environmental health hazards among countries have become greater. In areas near sources of pollution, particularly those with mixed residential and industrial activity and an economically disadvantaged population, residents are exposed to higher levels of air pollution (Finkelstein et al. 2005). This situation has aroused concerns about social injustice, and governments have been urged to take social inequality into account when considering air quality interventions. Studies in Europe and the United States have indicated a link between air pollution and poverty in terms of health impacts (Filleul et al. 2004; Schwartz 2000; Zanobetti and Schwartz 2000). In the Asia Pacific region, where air pollution and the burden of potentially avoidable morbidity and mortality are increasing (HEI 2004), no study has examined the interaction between socioeconomic status and pollution-related health outcomes.

The biologic mechanisms underlying the health effects of air pollution can be explained in terms of oxidative stress and immune system damage after both long- and short-term exposures. There are two main hypotheses regarding the possible effect of the interactions between air pollution and socioeconomic status on health. First, people of lower socioeconomic status are more likely to live and work in places with more toxic pollution. An alternative hypothesis is that because of inadequate access to medical care, lack of material resources, poorer nutrition, and higher smoking prevalence, those of lower socioeconomic status may be more susceptible to the adverse effects of air pollution than those in higher socioeconomic groups (O’Neill et al. 2003).

Health effects associated with socioeconomic factors can be assessed at both the individual and neighborhood levels according to an individual’s area of residence. The effect modification of air pollution by socioeconomic status measured at the individual level has been demonstrated in several epidemiologic studies (Filleul et al. 2004; HEI 2000; Krewski et al. 2005). However, the possible modification of air pollution effects associated with socioeconomic status, assessed at the neighborhood level, has not been well studied, and findings are still controversial (O’Neill et al. 2003). Whether residence in socially deprived areas is a greater environmental health hazard compared with residence in better-off areas is an important public health issue, and the possible effects need to be examined through appropriately designed studies.

Hong Kong is an affluent area in the Asia Pacific region, but poverty is still a problem among some subgroups of the population, resulting in serious social inequity. Socially deprived areas should be identified for additional community environmental protection and health resource allocation. Socioeconomic factors are usually multidimensional, and some of them, such as low income and low education, may be correlated with each other. Instead of studying several factors individually, we used a deprivation score at a specific community planning unit level to estimate neighborhood social deprivation for each of the subjects based on geographic code of their residency at the time of death, then assessed whether residents in poorer areas were subject to greater risk of mortality from ambient air pollution.

## Materials and Methods

### Tertiary planning units (TPUs)

The TPU system was devised by the Hong Kong Planning Department for town planning purposes. In 2001, the whole land area of Hong Kong was divided into 276 TPUs. Our analysis included all TPUs except for suburban TPUs (*n* = 67) in the New Territories and outer islands of Hong Kong, which are remote and have population densities lower than the lowest quartile (533/km^2^) of the whole territory. People residing in these sparsely populated areas account for about 1.5% of the total population and are usually exposed to sources and levels of air pollution different from those in urban areas. Because air pollution exposure measurements were based on data from monitoring stations located in urban areas, exclusion of nonurban areas would reduce exposure measurement errors.

### Measures of social deprivation

The Census and Statistics Department of Hong Kong conducts a population census every 10 years and a by-census every intermediate 5 years. TPUs are the smallest units in the population census report. The 2001 census report contains 44 statistics of the Hong Kong population measured at TPU level. We performed factor analysis on 18 socioeconomic and demographic variables related to social deprivation available in this population census database. Six factors accounting for 69% of the variation were extracted from principal-component analysis. Based on the distribution of factor loadings, we chose six variables to describe the conditions of social deprivation for each TPU: the proportions of the population with *a*) unemployment, *b* ) monthly household income < US$250, *c*) no schooling at all, *d* ) one-person household, *e*) never-married status, and *f*) subtenancy. Each of these six variables had significant factor loading for a specific principal factor, and all of them are deemed to be representative indicators of social disadvantage in the published literature and in the setting of the Hong Kong population. The first four conditions are more or less related to a lack of material resources. Being unmarried in Chinese society would have been regarded previously as undesirable in a social and family context. In Hong Kong, people who cannot afford to rent a whole flat may rent a part (usually a small room) of a flat from another tenant. The six selected variables in this study are similar to those used in other well-known social deprivation indices in other countries such as Index of Local Conditions (Department of Environment 1994) and the Jarman (Jarman 1983), and Townsend (Benach et al. 2001; Payne et al. 1996; Townsend et al. 1988) indices. For example, the “unemployment proportion” is similar to “unemployment rate”; “subtenancy” is similar to “not owner-occupier households”; “never married” is a dimension similar to “lone parent household”; “one-person household” could indicate partly “lone pensioner”; and “no school” is broadly similar to “low secondary education attainment” (Benach et al. 2001; Payne et al. 1996).

The social deprivation index (SDI) for each TPU was calculated by taking the average of these six selected variables. A detailed description of the development of SDI is given in one of our previous studies (Wong et al. 1999), which showed that each of these six measures was correlated with standard mortality rate at TPU level and mortality was high in TPUs with high SDI. Based on tertiles of SDI, all TPUs were classified into one of three SDI groups: low (less than the lowest tertile of SDI), middle (the lowest tertile to the middle tertile), and high (greater than the highest tertile). Table 1 shows a summary of basic characteristics for the 209 urban TPUs by SDI level.

### Health outcomes

The Census and Statistics Department of Hong Kong provided mortality data for all registered deaths from January 1996 to December 2002, including age, sex, date of death, TPU of residence, and the code of underlying cause of death, which is classified according to the *International Classification of Diseases, 9th Revision* (ICD-9), 1996–1999 and *10th Revision* (ICD-10), 2000–2002 (World Health Organization 1977, 1992). For each SDI group, we aggregated daily numbers of deaths due to all nonaccidental causes (ICD-9 codes 001-799; ICD-10 codes A00-T99, Z00-Z99), cardiovascular (ICD-9 390-459; ICD-10 I00-I99) and respiratory (ICD-9 460-519; ICD-10 J00-J98) diseases, respectively.

### Air pollution and meteorologic data

Hourly concentrations of nitrogen dioxide, sulfur dioxide, particulate matter with aero-dynamic diameter < 10 μm (PM_10_), and ozone were derived from eight fixed-site general monitoring stations operated by the Environmental Protection Department (HK EPD 2007). The measurement methods for NO_2_, SO_2_, PM_10_, and O_3_ were chemiluminescence, fluorescence, tapered element oscillating microbalance, and ultraviolet absorption, respectively. NO_2_, SO_2_, and O_3_ were also measured by differential optical absorption spectroscopy in some monitoring stations. Daily concentrations of air pollutants for each monitoring station were taken to be the average of the 24-hr concentrations of NO_2_, SO_2_, and PM_10_ and of 8-hr (0100–1800 hours) concentrations of O_3_. Daily concentrations of air pollutants for the whole territory of Hong Kong were evaluated by averaging the daily concentrations across all monitoring stations using the method of centering (Wong et al. 2001). In calculating the daily data there should be at least 75% 1-hr values of that particular day, and for each monitoring station there should be at least 75% of daily data complete for the whole study period. Meteorologic data, including daily temperature and relative humidity, were provided by the Hong Kong Observatory (2007).

### Statistical methods

We used generalized linear modeling to obtain the most adequate core models for each health outcome. We used Poisson regression with quasi-likelihood method to model mortality and hospital admission counts with adjustment for over-dispersion (McCullagh and Nelder 1989). To control for systematic variation over time, we introduced a trend and seasonality term and dummy variables for day of the week and public holidays. Other covariates considered and adjusted for were daily mean temperature and relative humidity. The trend and seasonality term was defined by fitting a natural smoothing spline with 4–6 degrees of freedom (dfs) per year. Additional smoothing splines with 3 dfs were included to adjust for the effects of temperature and 3 dfs to adjust for relative humidity. The choice of the number of dfs for each smoothing function was made on the basis of observed autocorrelations for the residuals using partial autocorrelation function plots. Partial autocorrelation coefficient (Hastie and Tibshirani 1990) of |ρ| < 0.1 for the first 2 lag days was used as a criterion for a minimally adequate model. Randomness of residuals and autoregressive terms were also considered in selecting the most appropriate models. If the above criteria were met, the variable for the air pollutant concentrations was entered into the core model for assessment of percentage excess risk (ER) per 10-μg/m^3^ increase of an air pollutant at single lag 0–4 days and at average lag 0 and lag 1 day. We performed Poisson regression analysis and assessed the ER for each level of social deprivation in the data set stratified by level of social deprivation. All analyses under Poisson regression were performed using the statistical software package R version 2.5.1 (R Development Core team 2006) with mgcv package version 1.3-25.

In addition, we used a case-only approach in a combined data set to assess potential interaction between social deprivation level and ambient air pollution on mortality. The case-only approach with logistic regression was originally proposed for studying the gene–environment interaction and has been widely used in this field of study (Fallin et al. 2003; Fracanzani et al. 2005). Armstrong (2003) has pointed out that this method can be extended for evaluating the interaction between time-varying variables and individual factors. Subsequently, Schwartz (2005) gave a more detailed description of this method and applied it to examine whether medical conditions modify the mortality effects of extreme temperature. We used this method recently to examine the effect modification of air pollution by individual smoking status and physical activity (Wong et al. 2007a, 2007b). In the present study, we assume that the risk of dying associated with temporary increase in air pollution level is modified by residence in different social deprivation areas. For example, people who died on days with high levels of air pollution would be more likely to reside in a high SDI area than those who died on days with low levels of air pollution, and therefore the air pollution level at the date of death could be a predictor of neighborhood SDI level of the deceased using logistic regression. The difference in relative risk of mortality associated with air pollution between SDI levels was calculated based on the relationship between SDI and the levels of ambient air pollution using multinomial logistic regression. Furthermore, an ordinal logit model was fitted to determine whether there was a trend in the health effects of air pollution increasing from low to middle and then to high SDI levels.

## Results

**Figure 1:**
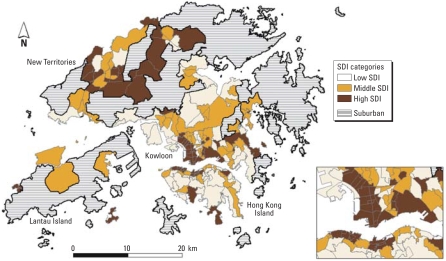
SDI in three levels for Hong Kong, 2001, excluding suburban areas.

Figure 1 shows the geographic variations in social deprivation in the whole of Hong Kong excluding suburban areas. Most of the areas with high SDI levels were in the northern territories bordering mainland China and in the outer islands. There were also a few highly deprived areas in the inner city.

### Health outcomes and covariates

Our study included a total of 215,240 nonaccidental deaths (males: 120,262; females: 94,978) from 1996 to 2002, with an average of 30,749 deaths per year. Summary statistics were compiled for daily counts of deaths from nonaccidental causes and from cardiovascular and respiratory diseases as well as daily meteorologic conditions and concentrations of the four air pollutants under study (Table 1). On each day there were, on average, 19, 36, and 17 deaths from non-accidental causes in the TPUs among low, middle, and high SDI levels, respectively.

### Effects of air pollution for all areas

In all areas, for nonaccidental and subcategory cardiovascular causes of mortality, the biggest single-day associations with all air pollutants occurred at either lag 0 or lag 1 day (Tables 2 and 3), but for subcategory respiratory mortality, they occurred at lag 2 day except with SO_2_, which occurred at lag 0 day (Table 4). There were statistically significant (*p* < 0.05) ERs for all the pollutants except O_3_ on all the three mortality outcomes.

### Separate effects of air pollution for each SDI group

**Figure 2:**
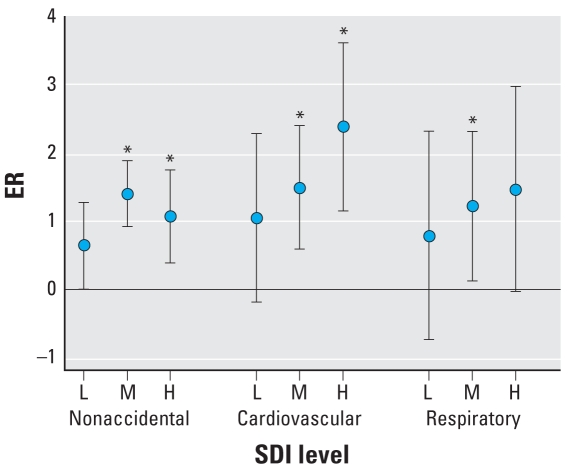
ER of mortality from nonaccidental, cardiovascular, and respiratory per 10-μg/m^3^ increase in NO_2_ concentration by three levels [low (L), middle (M), and high (H)] of social deprivation at average 0–1 lag day. Error bars indicate 95% CIs of estimates of ER. *p < 0.05.

**Figure 3:**
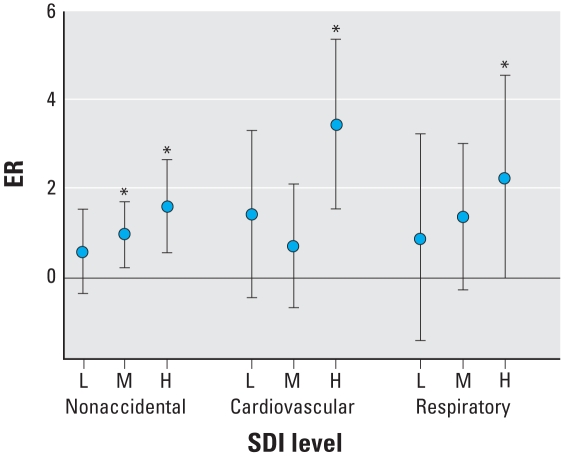
ER of mortality from nonaccidental, cardiovascular, and respiratory per 10 μg/m^3^ increase in SO_2_ concentration by three levels [low (L), middle (M), and high (H)] of social deprivation at average 0–1 lag day. Error bars indicate 95% CIs of estimates of ER. *p < 0.05.

The lag patterns of ER were comparable in the high, middle, and low SDI groups (Table 2). At average 0–1 lag—that is, with average pollutant concentration measured in the lag 0–1 day period—for NO_2_ and SO_2_, the point estimates of ER were higher in the middle SDI than in the low SDI group, except for SO_2_ for cardiovascular mortality, and were the highest in the high SDI group, except for NO_2_ for nonaccidental mortality (Figure 2). At average 0–1 lag, for PM_10_ and O_3_ the point estimates of ER were higher in the middle SDI than in the low SDI group (data not shown). Those in the high SDI group were higher than in the low SDI group (except the effect of PM_10_ on nonaccidental mortality). For respiratory mortality, at average 0–1 lag, for NO_2_ and SO_2_ the point estimates of ER increased from low to high SDI groups (Figures 2 and 3), with ER increasing from 0.76 to 1.44% for NO_2_ (Figure 2), and from 0.90 to 2.27% for SO_2_ (Figure 3). However, for PM_10_ and O_3_, the point estimates of ER varied from low to high SDI groups by only a small magnitude (0.82 to 0.70% for PM_10_; 0.23 to 0.0% for O_3_) (data not shown).

### Differences in effects of air pollution between SDI groups

The biggest difference in ER between SDI groups generally occurred at lag 1 day (data not shown). For nonaccidental mortality and for the subcategory cardiovascular mortality, the ER due to NO_2_ and SO_2_ at lag 1 day was significantly higher (*p* < 0.05) in the high SDI group than in the middle or low SDI groups; and the trends from low to high SDI groups were significant (*p* < 0.05) (data not shown). At the average 0–1 lag of a pollutant per 10 μg/m^3^, significantly (*p* < 0.05) greater ER for nonaccidental mortality, between high and middle SDI groups [change in ER 1.15%; 95% confidence interval (CI) 0.06–2.26] and between high and low (change in ER 1.38%; 95% CI, 0.13–2.63) SDI groups were shown (Table 5). Significant trend (change in ER 0.45%; 95% CI, 0.03–0.87) with change between middle and low or between high and middle SDI groups were found for an increase in concentrations of SO_2_, but not in concentrations of the other pollutants, although the differences in ER were in the same direction as that for SO_2_. For effects on cardiovascular mortality, significant increases (*p* < 0.05) in ER were found for SO_2_ (between high and middle SDI groups) and for NO_2_ (between high and low SDI groups); and significant trend (*p* < 0.05) was found for NO_2_. The magnitude of the difference and trend between SDI groups in effects of all pollutants on respiratory mortality were similar to those on all nonaccidental mortality but were statistically not significant (*p* > 0.05).

## Discussion

In Hong Kong, we found that air pollution mortality effects for SO_2_ were stronger in high compared with low SDI areas. Some previous studies in Hong Kong (Wong et al. 2001, 2002a) and Mainland China (Kan and Chen 2003; Venners et al. 2003; Xu et al. 1994, 1995) showed the gaseous pollutants NO_2_ and SO_2_ had stronger effects on morbidity and mortality compared with particulate air pollution in contrast to the findings in the United States (Samet et al. 2000). In this study, in addition to SO_2_ we found those residing in high SDI areas had higher ERs of death also associated with NO_2_, particularly for cardiovascular disease, than those in low SDI areas. A possible explanation is that socially deprived subgroups are more likely to have poorer health care and nutrition and other increased health risks, resulting in increased susceptibility to the adverse effects of air pollution. A meta-analysis of short-term health effects of air pollution (SO_2_, NO_2_, CO, PM_10_, and O_3_) in eight Italian cities showed that the ERs for hospital admission were modified by deprivation score and by NO_2_/PM_10_ ratio (Biggeri et al. 2005). Another explanation is that those residing in higher SDI areas may be exposed to higher levels of NO_2_ and SO_2_. A study in the Hamilton Census Metropolitan Area, Canada (Finkelstein et al. 2005), showed that subjects in the more deprived neighborhoods were exposed to higher levels of ambient particulates and gaseous pollutants. At least some of the observed social gradients associated with circulatory mortality arise from inequalities in environmental factors in terms of exposure to background and traffic-related pollutants. In Hong Kong, the daily levels of PM_10_ with correlations (*r*) between the eight monitoring stations ranged from 0.9 to 1.0 and annual average concentration from 42 to 55 μg/m^3^, indicating the homogeneity of PM_10_ exposure between SDI areas. However, the corresponding levels for NO_2_ ranged from 45 to 67 μg/m^3^ (*r* = 0.5–0.9), and 8–16 μg/m^3^ for SO_2_ (*r* = 0.4–0.8). The difference in the levels of NO_2_ and SO_2_ across geographic areas may partly explain the significant differences in their effects between SDI areas. On the other hand, in Hong Kong a large proportion of ambient air pollution is attributable to pollution emissions from road traffic (Wong et al. 2002b). Many deprived areas are located in the inner city on multiple busy traffic routes. Most of the population live next to roads and are affected by street canyon effects commonly formed by continuous building blocks in Hong Kong (Chan and Kwok 2000). In another study, high exposure to carbon monoxide was found to have a significant effect on asthma admissions for children 1–18 years of age, and the effect was greater for children with lower socioeconomic status (Neidell 2004).

In six regions of São Paulo City, Brazil, PM_10_ effects on daily respiratory deaths at the region level were negatively correlated with both the percentage of people with college education and high family income and were positively associated with the percentage of people living in slums, suggesting that social deprivation represents an effect modifier of the association between air pollution and respiratory deaths (Martins et al. 2004). In the city of Hamilton, Ontario, Canada, which was divided into five zones based on proximity to fixed-site air pollution monitors, SO_2_ and coefficient of haze (as a measure of particulate pollution) were associated with increased mortality, and the effects were higher among those zones with lower socioeconomic characteristics, lower educational attainment, and higher manufacturing employment (Jerrett et al. 2004).

There are several limitations to our study. First, we are aware that the SDI we defined may not reflect the whole profile of deprivation, although all of the information available from the census is included in the computation. Second, there may be heterogeneity within areas having the same SDI levels that have not been accounted for. However, we classified SDI levels into three broad categories, which should help reduce misclassification of deprivation. Third, population-level exposures using average concentrations from a limited number of air pollution monitors as a proxy for each individual may be subject to some measurement errors, and consequently we cannot determine whether the increased pollution-related mortality risk in high SDI areas is due mainly to greater pollutant exposure or increased biologic susceptibility. However, the population density in Hong Kong is very high (about 6,200/km^2^), and the daily air pollution levels among eight monitoring stations included in the study were highly correlated. This justifies our use of the average air pollution concentrations over all monitoring stations as daily concentrations for the whole territory. The aggregated daily concentrations derived for the whole of Hong Kong should be at least as reliable as measurements used in other daily time-series air pollution studies. In this study, we used PM_10_ to assess the effect of particulate pollution, because the measurements of PM_2.5_ were not available in all the stations under study during the period of the study. However, based on the available data from two stations, the Spearman correlation coefficient between daily levels of the two measures was 0.89, and PM_2.5_ constituted a high proportion of PM_10_ (around 70%); therefore, it is unlikely that estimates using the two measures would differ to a great extent in Hong Kong. Unlike specific gaseous pollutants that are comparable from place to place, the potency of PM_10_ will depend on the composition of the particulates, which may vary greatly in different geographic locations. The comparability of air pollution studies on health effects of particulates may be related more to specific subspecies than the particle size measured. Finally, the mechanisms underlying why some population groups with high SDI experienced higher adverse effects of air pollution are still unclear, and research on specific protective interventions is needed.

## Conclusions

This study provides evidence that neighborhood socioeconomic status plays a role in the association between ambient air pollution and mortality. Residence in areas of high social deprivation may increase the mortality risks associated with air pollution. These findings should promote discussion among scientists, policy makers, and the public about social inequities in health when considering environmental protection and management in the context of economic, urban, and infrastructural development.

## Figures and Tables

**Table 1:** Summary statistics for TPUs by three levels of social deprivation, air pollution, and meteorologic variables for whole territories.

Variable	Min	1st Quartile	Median	3rd Quartile	Max	Mean	SD
Population size (× 10,000)							
Low SDI	0.40	1.19	2.32	5.75	18.99	4.22	4.19
Middle SDI	0.12	1.05	4.86	7.11	20.36	5.25	4.86
High SDI	0.11	0.76	1.42	2.52	8.63	2.07	1.99
Area (km^2^)							
Low SDI	0.13	0.83	1.82	4.54	14.08	3.33	3.45
Middle SDI	0.13	0.81	1.62	3.05	35.61	3.43	6.37
High SDI	0.06	0.38	0.79	2.43	16.30	2.56	4.00
Population density (× 10,000/km^2^)							
Low SDI	0.09	0.55	1.68	3.80	16.75	2.49	2.76
Middle SDI	0.04	0.46	3.06	6.40	15.48	4.23	4.03
High SDI	0.05	0.28	2.52	6.02	17.95	3.75	4.14
Mortality (daily count)							
Low SDI	5.0	16.0	19.0	23.0	46.0	19.3	5.3
Middle SDI	13.0	31.0	36.0	42.0	66.0	36.2	8.0
High SDI	3.0	13.0	17.0	21.0	40.0	17.4	5.4
Air pollutants (μg/m^3^)							
NO_2_	10.1	45.1	56.3	69.6	168.0	58.7	20.0
SO_2_	1.8	9.6	14.7	22.1	109.4	17.8	12.1
PM_10_	13.5	31.8	45.5	66.7	188.5	51.6	25.3
O_3_	−8.2	19.2	31.7	50.8	196.6	36.9	23.0
Temperature (°C)	6.9	19.8	24.7	27.8	33.8	23.7	4.9
Relative humidity (%)	27.0	74.0	79.0	84.0	97.0	77.9	10.0

Abbreviations: Max, maximum; Min, minimum.

**Table 2:** Excess risk (%) of nonaccidental mortality per 10-μg/m^3^ increase in pollutant concentration by three levels of social deprivation at lag 0, 1, 2, 3, and 4 days.

	Lag	Low SDI ER (95% CI)	Middle SDI ER (95% CI)	High SDI ER (95% CI)	All areas ER (95% CI)
NO_2_	0	0.55 (0.00 to 1.11)	1.07 (0.65 to 1.50)	0.53 (−0.06 to 1.13)	0.75 (0.45 to 1.06)
	1	0.40 (−0.15 to 0.95)	1.04 (0.61 to 1.46)	1.07 (0.48 to 1.66)	0.79 (0.49 to 1.10)
	2	0.16 (−0.37 to 0.70)	0.62 (0.21 to 1.04)	0.52 (−0.05 to 1.10)	0.37 (0.07 to 0.67)
	3	0.29 (−0.24 to 0.82)	0.39 (−0.03 to 0.80)	0.12 (−0.45 to 0.70)	0.20 (−0.10 to 0.50)
	4	−0.30 (−0.82 to 0.24)	0.12 (−0.29 to 0.53)	−0.22 (−0.79 to 0.36)	−0.12 (−0.41 to 0.18)
SO_2_	0	0.64 (−0.16 to 1.44)	0.76 (0.14 to 1.38)	0.81 (−0.05 to 1.68)	0.68 (0.24 to 1.12)
	1	0.21 (−0.57 to 1.00)	0.62 (0.02 to 1.23)	1.44 (0.60 to 2.29)	0.62 (0.19 to 1.06)
	2	0.23 (−0.53 to 1.01)	0.34 (−0.26 to 0.93)	0.33 (−0.50 to 1.17)	0.20 (−0.23 to 0.63)
	3	0.15 (−0.61 to 0.92)	0.14 (−0.45 to 0.74)	−0.45 (−1.28 to 0.38)	−0.10 (−0.53 to 0.32)
	4	−0.70 (−1.46 to 0.07)	0.18 (−0.41 to 0.77)	−0.55 (−1.38 to 0.28)	−0.24 (−0.66 to 0.18)
PM_10_	0	0.37 (−0.10 to 0.84)	0.70 (0.34 to 1.07)	0.22 (−0.29 to 0.73)	0.45 (0.19 to 0.72)
	1	0.40 (−0.04 to 0.84)	0.48 (0.14 to 0.82)	0.46 (−0.01 to 0.94)	0.40 (0.15 to 0.64)
	2	0.14 (−0.28 to 0.57)	0.35 (0.02 to 0.68)	0.29 (−0.17 to 0.75)	0.22 (−0.02 to 0.45)
	3	−0.12 (−0.55 to 0.30)	0.18 (−0.14 to 0.51)	−0.05 (−0.51 to 0.40)	0.00 (−0.24 to 0.23)
	4	−0.14 (−0.56 to 0.28)	0.17 (−0.16 to 0.50)	−0.06 (−0.51 to 0.40)	0.03 (−0.20 to 0.26)
O_3_	0	−0.20 (−0.73 to 0.34)	0.41 (0.00 to 0.82)	0.53 (−0.04 to 1.11)	0.23 (−0.07 to 0.52)
	1	0.22 (−0.26 to 0.70)	0.46 (0.09 to 0.83)	0.02 (−0.49 to 0.54)	0.27 (0.00 to 0.53)
	2	0.20 (−0.25 to 0.65)	0.23 (−0.12 to 0.58)	0.19 (−0.30 to 0.68)	0.18 (−0.07 to 0.43)
	3	0.00 (−0.44 to 0.45)	0.21 (−0.14 to 0.55)	0.18 (−0.30 to 0.66)	0.13 (−0.11 to 0.38)
	4	−0.17 (−0.60 to 0.27)	0.04 (−0.29 to 0.38)	−0.03 (−0.50 to 0.45)	−0.02 (−0.27 to 0.22)

**Table 3:** Excess risk (%) of cardiovascular mortality per 10-μg/m^3^ increase in pollutant concentration by three levels of social deprivation at lag 0, 1, 2, 3, and 4 days.

	Lag	Low SDI ER (95% CI)	Middle SDI ER (95% CI)	High SDI ER (95% CI)	All areas ER (95% CI)
NO_2_	0	0.82 (−0.25 to 1.90)	1.24 (0.45 to 2.03)	1.45 (0.37 to 2.53)	1.17 (0.61 to 1.73)
	1	0.76 (−0.30 to 1.83)	1.00 (0.22 to 1.78)	2.14 (1.07 to 3.21)	1.08 (0.53 to 1.64)
	2	0.34 (−0.70 to 1.39)	0.85 (0.08 to 1.61)	0.95 (−0.09 to 2.00)	0.53 (−0.02 to 1.08)
	3	0.27 (−0.76 to 1.31)	0.46 (−0.30 to 1.23)	−0.28 (−1.32 to 0.77)	0.09 (−0.45 to 0.63)
	4	−0.51 (−1.54 to 0.52)	0.08 (−0.67 to 0.84)	0.02 (−1.01 to 1.06)	−0.13 (−0.66 to 0.41)
SO_2_	0	1.10 (−0.45 to 2.68)	0.71 (−0.44 to 1.87)	1.85 (0.28 to 3.44)	1.03 (0.21 to 1.85)
	1	0.89 (−0.64 to 2.44)	0.30 (−0.83 to 1.45)	2.88 (1.35 to 4.43)	0.93 (0.13 to 1.74)
	2	0.38 (−1.12 to 1.90)	0.36 (−0.75 to 1.48)	1.28 (−0.22 to 2.81)	0.42 (−0.37 to 1.21)
	3	0.26 (−1.23 to 1.77)	0.25 (−0.85 to 1.37)	0.06 (−1.45 to 1.58)	0.10 (−0.69 to 0.89)
	4	−0.75 (−2.24 to 0.76)	−0.27 (−1.36 to 0.85)	0.66 (−0.84 to 2.19)	−0.21 (−1.00 to 0.58)
PM_10_	0	0.14 (−0.77 to 1.06)	0.66 (0.00 to 1.34)	0.83 (−0.08 to 1.75)	0.52 (0.05 to 1.00)
	1	0.64 (−0.21 to 1.49)	0.49 (−0.13 to 1.12)	0.89 (0.04 to 1.75)	0.58 (0.14 to 1.03)
	2	0.24 (−0.58 to 1.07)	0.80 (0.20 to 1.40)	0.12 (−0.70 to 0.95)	0.43 (0.00 to 0.86)
	3	−0.27 (−1.09 to 0.55)	0.65 (0.06 to 1.25)	−0.09 (−0.91 to 0.73)	0.14 (−0.28 to 0.57)
	4	0.01 (−0.80 to 0.83)	0.52 (−0.07 to 1.12)	0.04 (−0.77 to 0.86)	0.23 (−0.20 to 0.65)
O_3_	0	0.23 (−0.81 to 1.29)	0.57 (−0.19 to 1.35)	0.66 (−0.39 to 1.72)	0.42 (−0.12 to 0.97)
	1	0.41 (−0.53 to 1.35)	0.65 (−0.04 to 1.34)	0.23 (−0.71 to 1.18)	0.45 (−0.04 to 0.94)
	2	0.51 (−0.37 to 1.40)	0.52 (−0.13 to 1.17)	0.23 (−0.66 to 1.13)	0.38 (−0.08 to 0.84)
	3	0.51 (−0.35 to 1.39)	0.55 (−0.09 to 1.19)	−0.17 (−1.04 to 0.71)	0.28 (−0.17 to 0.74)
	4	−0.29 (−1.15 to 0.58)	0.02 (−0.61 to 0.66)	−0.51 (−1.37 to 0.37)	−0.23 (−0.68 to 0.22)

**Table 4:** Excess risk (%) of respiratory mortality per 10-μg/m^3^ increase in pollutant concentration by three levels of social deprivation at lag 0, 1, 2, 3, and 4 days.

	Lag	Low SDI ER (95% CI)	Middle SDI ER (95% CI)	High SDI ER (95% CI)	All areas ER (95% CI)
NO_2_	0	1.02 (−0.31 to 2.36)	0.76 (−0.20 to 1.72)	0.97 (−0.34 to 2.30)	0.88 (0.19 to 1.58)
	1	0.16 (−1.16 to 1.49)	1.07 (0.13 to 2.03)	1.26 (−0.04 to 2.57)	0.90 (0.22 to 1.60)
	2	−0.05 (−1.34 to 1.26)	1.02 (0.10 to 1.96)	1.62 (0.35 to 2.91)	0.92 (0.25 to 1.60)
	3	0.13 (−1.16 to 1.43)	0.94 (0.02 to 1.87)	0.95 (−0.32 to 2.23)	0.75 (0.08 to 1.42)
	4	−0.53 (−1.81 to 0.77)	0.51 (−0.40 to 1.44)	−0.30 (−1.56 to 0.98)	0.05 (−0.62 to 0.72)
SO_2_	0	1.21 (−0.70 to 3.16)	0.57 (−0.80 to 1.95)	1.84 (−0.04 to 3.76)	1.06 (0.06 to 2.06)
	1	0.06 (−1.83 to 1.98)	1.33 (−0.01 to 2.68)	1.32 (−0.53 to 3.20)	1.02 (0.04 to 2.01)
	2	0.45 (−1.40 to 2.33)	1.01 (−0.31 to 2.34)	1.47 (−0.34 to 3.32)	0.99 (0.03 to 1.96)
	3	0.32 (−1.53 to 2.20)	1.30 (−0.01 to 2.62)	−0.67 (−2.48 to 1.18)	0.56 (−0.40 to 1.52)
	4	−1.36 (−3.21 to 0.53)	0.77 (−0.54 to 2.10)	−1.05 (−2.87 to 0.81)	−0.21 (−1.17 to 0.76)
PM_10_	0	0.69 (−0.44 to 1.82)	0.31 (−0.50 to 1.13)	0.27 (−0.85 to 1.40)	0.39 (−0.20 to 0.99)
	1	0.55 (−0.50 to 1.61)	0.77 (0.01 to 1.53)	0.72 (−0.32 to 1.78)	0.70 (0.15 to 1.26)
	2	0.36 (−0.66 to 1.39)	0.85 (0.12 to 1.59)	1.46 (0.45 to 2.47)	0.89 (0.36 to 1.42)
	3	−0.24 (−1.25 to 0.78)	0.66 (−0.07 to 1.39)	0.70 (−0.30 to 1.71)	0.45 (−0.08 to 0.98)
	4	−0.17 (−1.17 to 0.85)	0.69 (−0.03 to 1.42)	0.48 (−0.52 to 1.48)	0.43 (−0.10 to 0.96)
O_3_	0	−0.22 (−1.50 to 1.07)	0.02 (−0.90 to 0.94)	0.60 (−0.66 to 1.88)	0.11 (−0.55 to 0.79)
	1	0.46 (−0.68 to 1.61)	0.26 (−0.56 to 1.09)	−0.51 (−1.65 to 0.64)	0.11 (−0.48 to 0.72)
	2	−0.01 (−1.09 to 1.09)	0.50 (−0.28 to 1.28)	0.42 (−0.65 to 1.51)	0.36 (−0.21 to 0.93)
	3	−0.31 (−1.38 to 0.77)	0.24 (−0.52 to 1.01)	0.55 (−0.50 to 1.62)	0.19 (−0.37 to 0.75)
	4	−0.01 (−1.06 to 1.06)	0.04 (−0.71 to 0.80)	0.88 (−0.16 to 1.93)	0.25 (−0.30 to 0.80)

**Table 5:** Difference in ER [% (95% CI)] of mortality between areas with different SDI levels associated with air pollutants per 10-μg/m^3^ increase at average lag 0–1 day.

	Pollutant	Nonaccidental causes	Cardiovascular disease	Respiratory disease
High vs. middle	NO_2_	0.45 (−0.16 to 1.06)	1.03 (−0.11 to 2.18)	0.94 (−0.41 to 2.31)
	SO_2_	1.15 (0.06 to 2.26)	2.74 (0.66 to 4.85)	1.62 (−0.83 to 4.12)
	PM_10_	0.23 (−0.25 to 0.72)	0.49 (−0.40 to 1.40)	0.49 (−0.58 to 1.58)
	O_3_	0.14 (−0.41 to 0.70)	0.09 (−0.95 to 1.14)	0.75 (−0.50 to 2.01)
High vs. low	NO_2_	0.51 (−0.18 to 1.20)	1.35 (0.49 to 2.67)	0.59 (−0.98 to 2.18)
	SO_2_	1.38 (0.13 to 2.63)	2.16 (−0.19 to 4.57)	2.42 (−0.47 to 5.38)
	PM_10_	0.12 (−0.42 to 0.67)	0.82 (−0.20 to 1.86)	−0.15 (−1.39 to 1.10)
	O_3_	0.14 (−0.48 to 0.76)	0.13 (−1.06 to 1.33)	0.33 (−1.12 to 1.79)
Trend test	NO_2_	0.16 (−0.07 to 0.39)	0.45 (0.01 to 0.88)	0.21 (−0.32 to 0.73)
	SO_2_	0.45 (0.03 to 0.87)	0.71 (−0.08 to 1.51)	0.81 (−0.15 to 1.71)
	PM_10_	0.04 (−0.15 to 0.22)	0.27 (−0.07 to 0.61)	−0.04 (−0.46 to 0.37)
	O_3_	0.05 (−0.16 to 0.25)	0.04 (−0.35 to 0.44)	0.12 (−0.37 to 0.60)
